# The catalytic function of cytochrome P450 is entwined with its membrane-bound nature

**DOI:** 10.12688/f1000research.11015.1

**Published:** 2017-05-09

**Authors:** Carlo Barnaba, Katherine Gentry, Nirupama Sumangala, Ayyalusamy Ramamoorthy

**Affiliations:** 1 Biophysics Program and Department of Chemistry, The University of Michigan, Ann Arbor, MI, USA

**Keywords:** cytochrome P450, NADPH-P450 reductase, cytochrome b5, membrane, structure, sold-state NMR

## Abstract

Cytochrome P450, a family of monooxygenase enzymes, is organized as a catalytic metabolon, which requires enzymatic partners as well as environmental factors that tune its complex dynamic. P450 and its reducing counterparts—cytochrome P450-reductase and cytochrome *b _5_*—are membrane-bound proteins located in the cytosolic side of the endoplasmic reticulum. They are believed to dynamically associate to form functional complexes. Increasing experimental evidence signifies the role(s) played by both protein-protein and protein-lipid interactions in P450 catalytic function and efficiency. However, the biophysical challenges posed by their membrane-bound nature have severely limited high-resolution understanding of the molecular interfaces of these interactions. In this article, we provide an overview of the current knowledge on cytochrome P450, highlighting the environmental factors that are entwined with its metabolic function. Recent advances in structural biophysics are also discussed, setting up the bases for a new paradigm in the study of this important class of membrane-bound enzymes.

## Introduction

Cytochrome P450 (CYP) is a superfamily of heme-containing enzymes responsible for insertion of molecular oxygen into inactivated C-H and C-C bonds. CYPs are ubiquitous in human tissues, being responsible for the metabolism of drug and xenobiotic compounds ^1^; several CYPs are also involved in the biosynthesis of steroids ^2, 3^. In humans, 57 genes that code for various CYP enzymes were identified; about 13 isoforms are responsible for the metabolism of more than 80% of the clinically used drugs ^1^. Human CYPs that typically mediate metabolic clearance of drugs are targeted to the endoplasmic reticulum (ER) by an N-terminal domain that spans the membrane bilayer with the catalytic domain residing in the cytosol and partially embedded in the membrane ^1, 4^.

**Figure 1.**
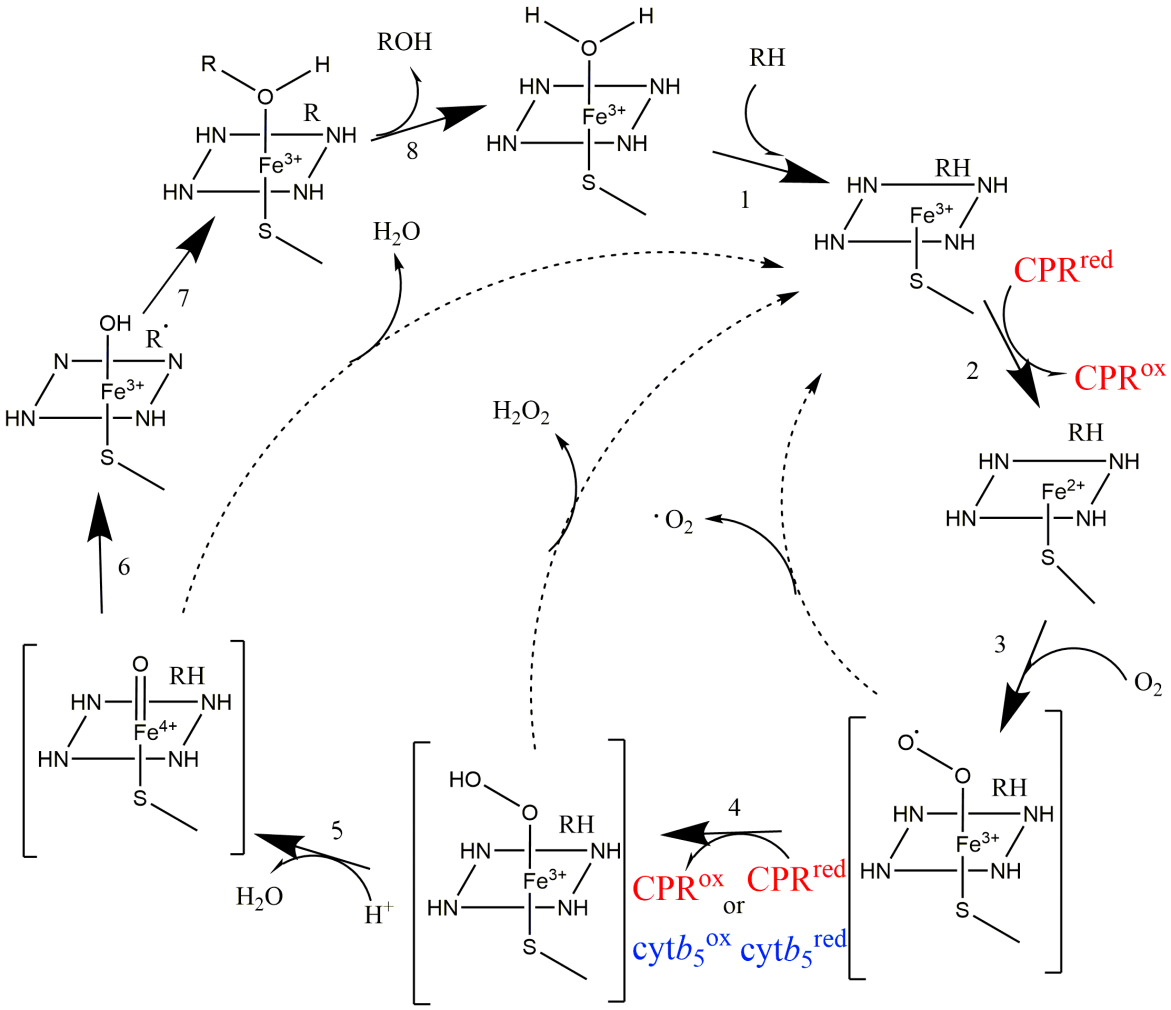
The catalytic cycle of cytochrome P450. The binding of substrate R-H (1) causes a decrease of the redox potential of about 100 mV, which allows the first electron transfer from cytochrome P450-reductase (CPR) (2). The reduction of Fe ^3+^ to Fe ^2+^ makes suitable O _2_ binding (3), which now can accept a second electron from either CPR or cytochrome *b _5_* (cyt *b _5_*) (4), to form a hydroperoxyl intermediate known as compound 0. The O _2_^−2^ complex reacts with surrounding protons to form the highly reactive oxyferryl intermediate, also known as compound I (5). The Fe-ligated O atom (6) is transferred to the substrate forming a hydroxylated form of the substrate (7). The product is finally released (8), replaced by a molecule of water. Three uncoupling reactions are shown as dashed lines, with the respective products: the autoxidation shunt O _2_^−2^, the peroxide shunt (H _2_O _2_), and the oxidase shunt (H _2_O).

**Figure 2.**
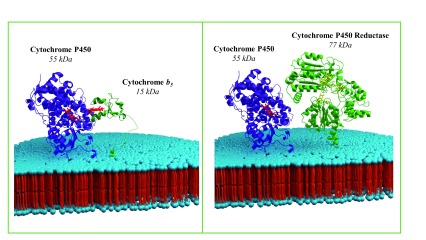
Model structures of cytochrome P450 with cytochrome *b _5_* (left) and cytochrome P450 with cytochrome P450 NADPH-reductase (right) in a lipid bilayer. The models were constructed from the crystallographic or solution-state nuclear magnetic resonance (NMR) structures of the soluble domains of the proteins. The structure, dynamics, and topology of the invisible transmembrane domains of the full-length cytochrome proteins have been determined for the first time by static solid-state NMR experiments on magnetically aligned bicelles. These studies reported that the helical structures of the transmembrane domains are significantly tilted away from the lipid bilayer normal and undergo motion in the millisecond (or slower) timescale that is far slower than that of the residues in the soluble domain ^17, 18, 45^.

For its catalytic function, CYP needs two electrons, provided by its redox partners, cytochrome P450-reductase (CPR) and cytochrome *b _5_* ( *b _5_*) ( Figure 1). CPR and *b _5_* are also found in the cytoplasmic side of the ER of eukaryotic cells and also possess a hydrophobic transmembrane binding domain (TM) ^5, 6^. CPR is a large flavoprotein (approximately 77 kDa), containing FAD and FMN domains, linked by a “hinge” region which provides conformational plasticity. On the other hand, *b _5_* is a small hemoprotein (15 kDa), which promotes catalysis not only via electron transfer, but also through allosteric regulation ^7^. A schematic of P450, CPR, and *b _5_* incorporated in a lipid bilayer is depicted in Figure 2.

The interactions between CYPs and the redox counterparts as well as their catalytic activities—including substrate binding and metabolite release—occur in the membrane landscape. The biphasic amphiphilic environment sets up the molecular requirements for cytochrome P450 metabolon, as observed since the dawn of P450 research ^8, 9^. Since then, efforts have been made to reconstitute CYP’s catalytic activity in membrane mimic systems, from simple binary ^9^ or ternary ^10^ lipid mixtures to the more sophisticated nanodiscs ^11, 12^, which consist of a lipid patch surrounded by a polypeptide or polymeric chain. More recently, P450 activity has been reconstituted in biomimetic of the ER to study protein-lipid interaction at the single-molecule level ^13^.

**Figure 3.**
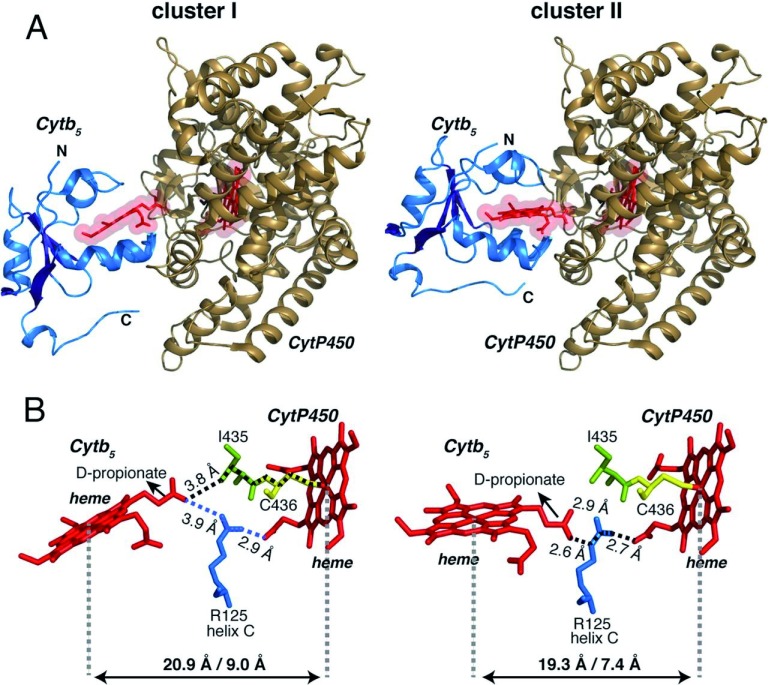
First high-resolution structure of the membrane-bound cytochromes-b5-P450 complex revealing electron transfer pathway. ( **A**) Three-dimensional structure of the membrane-bound rabbit cytochrome P4502B4–cytochrome *b _5_* complex reproduced from Ahuja *et al*. ^19^. It was obtained by using the high-resolution (solution- and solid-state) nuclear magnetic resonance (NMR) structure of membrane-bound rabbit cytochrome *b _5_* (PDB code is 2M33), crystal structure of the soluble domain of cytochrome P4502B4 (PDB code is 1SUO), and NMR and mutational constraints to obtain the structure of the membrane-bound complex. ( **B**) Electron transfer pathway revealed by HARLEM ^63^ in the complex structure. PDB, Protein Data Bank.

Paradoxically, if much is known about the effects of proteins and lipids turning on CYP function from an ensemble perspective, the molecular details of these protein-protein and protein-lipid interactions are mostly unexplored ^14^. The intrinsic properties of the cell membrane and the innumerous interactions among molecules—amphipathic lipids, polysaccharides, cholesterol, proteins, and water—provide paramount challenges for biophysics ^15^. Traditionally, several structures of truncated versions of CYPs have been elucidated at high resolution by x-ray crystallography; CPR and *b _5_* have also been resolved ^5, 16^. In all of these structures, the membrane binding domain has been cleaved to allow complete solubility and subsequent crystallization; the lipids were not present in these structures. As a result, the published structures are devoid of any information pertaining to the molecular organization of these enzymes in the membrane. Nuclear magnetic resonance (NMR) spectroscopy has played a pivotal role in the structure determination of biomacromolecules, providing scientists with detailed structural and dynamical information that is inaccessible through other biophysical means ^15^. In this respect, NMR spectroscopy is both an alternative and a complement to x-ray crystallography. In the last decade, both solution- and solid-state NMR spectroscopy have offered new and detailed insights into the structure, dynamics, and topology of cytochrome *b _5_* in lipid bilayers and its interface with P450 ( Figure 3) ^6, 17– 19^.

This short review will discuss several catalytic aspects of cytochrome P450 machinery that are intrinsically linked to their membrane-bound nature. We will underline novel mechanistic insights that structural NMR and other biophysical tools have provided, stressing the need for a new paradigm in the structural studies of this important class of membrane-bound enzymes.

## Membrane and membrane-anchor domains modulate P450 metabolism and its catalytic efficiency

CYP-mediated oxidation of drugs and xenobiotic in the ER is made possible by the sequential donation of two electrons by CPR and *b _5_* ^20^. The electron transfer is believed to occur through the dynamic interactions between these proteins. For the electron to be shuttled, it needs to overcome a redox potential gradient. As depicted in Figure 1, *b _5_* is unable to donate the first electron since it cannot overcome the redox potential barrier. The composition of phospholipids can modulate the midpoint potentials for both CYPs and CPR, as demonstrated by Das and Sligar using nanodiscs ^21^. When CPR was incorporated in nanodiscs, the redox potentials of both the FAD and FMN domains were shifted to more positive values, compared with CPR lacking the TM domain. Also, the presence of anionic phospholipids made the redox potential suitable for electron transfer from CPR to P450 ^21^, a result that confirmed kinetics observations made by other groups ^22^. The catalytic activity and membrane insertion of CYP3A4, which metabolize more than 50% of commercial drugs ^23^, increased as a function of anionic phospholipid concentration ^22^. It is recognized that membrane composition and polarity can modify both V _max_ and *K _M_* ^22, 24^, which are respectively a measure of catalytic rate and binding affinity under the steady-state assumption of the Michaelis-Menten equation. Phospholipid composition has also been related to protein folding and stability, as reported by Jang *et al*. for full-length and TM-truncated CYP1B1 ^24^, as well as CYP3A4 ^25^. In regard to the putative role of *b _5_* as electron donor and allosteric effector for CYP-mediated drug clearance, it is well known that it is isoform-dependent. Allosteric modulations have been observed for CYP3A4 hydroxylation of testosterone and nifedipine ^26^ but not for CYP1A1 and CYP2D6 1′-hydroxylation of bufuralol ^27^. Deletion of amino acids of the TM α-helix of rabbit *b _5_* decreased the binding affinity for CYP2B4 by several folds, as well as the catalytic turnover ^28^.

The efficiency of cytochrome P450 catalysis is measured in terms of “coupling”, meaning the amount of transferred electron that is committed to the monooxygenase activity versus the extent of the “uncoupled” (or unproductive) pathways. Three unproductive pathways exist ( Figure 1): (a) release of superoxide from the ferrous dioxygen, (b) release of hydrogen peroxide, and (c) the 4e ^−^ reduction of heme to produce water ^20^. Recent studies have postulated that both TM domains *and* the nature of the membrane may have a role in the coupling efficiency. McDougle *et al*. ^29^ found a correlation between the coupling efficiencies of CYP2J2 with the length of the TM domain of CPR: the more extensive the deletion of the TM helix, the lesser the coupling efficiencies. Similar results have been reported for CYP2C19 coupling with several substrates: full-length versus truncated CPR, as well as the presence or absence (or both) of lipids, was related to the overall catalytic efficiency ^30^. Grinkova *et al*. ^31^ observed an increase of coupling when CYP3A4 was incorporated in nanodiscs containing a higher amount of anionic phospholipids, which the authors attributed to the changes in the redox potential of CYP3A4 and reductase. On the P450 side, it is believed that the TM domain of CYPs serves as an “anchor” to the membrane, playing a pivotal role in protein orientation. In CYP1A2 and CYP2D6, the hydrophobicity of the TM domain has been shown to regulate the efficiency of interaction of CYPs with CPR or phospholipids or both and therefore their catalytic activities ^32– 35^. Similar results have been reported for CYP1B1, and decreased catalytic activity was observed when the TM domain was partially cleaved ^24^.

Specific interactions between the P450 metabolon and the membrane are believed to play a major role in this catalytic modulation. Traditional x-ray crystallographic studies on P450-CPR and P450- *b _5_* complexes provided snapshots of their quaternary organization and have been accompanied by specific amino acid mutations able to disrupt/enhance functionality ^4, 36– 38^. Recently, solution NMR experiments have complemented those initial efforts, providing realistic insights of the complex interface as well as of ligand-induced modifications ^39^. Other studies have explored the potential of this technique for studying protein complexes between the steroidogenic CYP17A1 and both *b _5_* and the FMN domain of CPR in the absence of membrane ^3, 14^. However, complete solubilization and subsequent crystallization of protein-protein complexes have been obtained only by removing the 60-residue segment containing the hydrophobic transmembrane domain of the proteins. Since membrane was not present in these structure studies of protein-protein complexes, the reported results do not contain any information about the structure and topology of the transmembrane domains of these proteins.

Our lab is the first in the field to have used both solution- and solid-state NMR (ssNMR) techniques in studying P450 and related proteins ^6^ in a functional amphiphilic bilayer, relying on the capacity of both bicelles ^15, 40^ and nanodiscs ^41^ to incorporate and preserve the functionality of these protein. The high-resolution structure of the membrane-bound cytochrome-P450- *b _5_* complex is shown in Figure 3 ^19^. Solid-state NMR experiments on magnetically aligned bicelles revealed the presence of transmembrane helix and its topology in CYP2B4, CPR, and *b _5_* and further revealed the significant difference in the timescales of motion of residues in the soluble linker and TM domains of *b _5_* that is crucial in the formation of the productive protein-protein complex ^17, 42^. Studying isotopically labeled *b _5_* in bicelles by ssNMR has shown how the mobility of the TM domain of *b _5_* is significantly reduced by the presence of CYP2B4 without altering its geometry and helical structure ^17^. In addition, ssNMR experiments on aligned bicelles revealed the direct interactions between the TM domains of CYP2B4 and *b _5_*, in which the “leucine zipper” in the TM domain of *b _5_* plays a very important role. More importantly, NMR confirmed the electrostatic nature of the *b _5_*-CYP2B4 interactions and that the presence of substrate promotes specific interactions between the two proteins ^43^. CYP2B4- *b _5_* complex was more recently studied in nanodiscs, in a detergent-free environment that better simulates the native membrane ^41^, obtaining high-resolution structural interactions between the proteins. In addition, interactions between membrane-bound CYP2B4, *b _5_*, and CPR have been measured with and without the presence of ligands by using high-resolution NMR experiments ^44^. The FMN-binding domain (FBD) of CPR has also been studied in bicelles in solid-state NMR experiments ^45^. Our group was also able to characterize for the first time the interplay among proteins in a tertiary FBD-P450- *b _5_* complex by NMR spectroscopy in lipid bilayers ^44^. Notably, we showed that substrate binding to P450 can disrupt P450-CPR complex, facilitating the association of *b _5_*, and that the extent of this equilibrium shift is highly dependent on the substrate. This result can potentially explain the observed difference in the catalytic modulation provided by *b _5_*.

In addition, the structural interaction of FBD with cytochrome *c*—a small hemoprotein essential for the electron transport chain in mitochondria—has been reported by using cytochrome *c* as an efficient model system to test the electron transfer process ^46^. Solution NMR titration experiments showed the formation of a dynamic complex between the two proteins, on a fast exchange timescale. NMR restraints were implemented in molecular docking to generate structural models and map the binding interface on the FBD. The proposed structural model of the FBD–cytochrome *c* complex suggested potential electron pathways that provide strong electronic coupling between the redox centers ^46^. More high-resolution structure-based functional and dynamical studies on the binary and ternary P450-redox complexes and their interactions with drugs are in progress in our lab.

## Access path channels, substrate availability, and membrane partitioning

As from the above discussion, the membrane and the membrane domains can play a substantial role in P450 catalysis, modulating both electron transfer between the redox couple(s), as well as the overall efficiency of the catalysis. The membrane is also a thick interface between the surroundings (that is, cytosol) and CYPs. Indeed, it has been shown that the membrane can slow down the access of water as well as substrate to the active site ^47^. Also, lipophilic compounds that are poorly soluble are predominantly partitioning in the membrane, allowing P450 recruiting of hydrophobic substrates directly from the lipid phase ^21, 48, 49^. For example, Murtazina *et al*. ^50^ demonstrated that CYP27A1 affinities for 5α-cholestane-3α,7α,12α-triol and cholesterol were decreased in the presence of the negatively charged phosphatidylglycerol, which can potentially reflect either a different partitioning of these steroidal molecules or an easier access to substrate due to protein/membrane interactions. Differences in the binding affinities and spin equilibrium in soluble versus membrane-anchored P450 have already been reported ^25, 49, 51– 53^, and their significance is relevant given how crucial the affinity parameters are for pharmacokinetic/pharmacodynamic models ^54^. In this regard, Denisov *et al*. ^49^ have demonstrated the presence of an allosteric site at the membrane interface of CYP3A4, emphasizing how crucial the presence of membrane is for the evaluation of drug-drug interactions in pharmacological studies. In addition, recent studies from Atkins’s group have provided experimental insights into substrate modulation of CYP3A4 when in nanodiscs ^25, 53^. The embedded portion of the protein can dynamically interact with the membrane, allowing the opening of substrate channels and a water “aqueduct” ^25^. These properties—along with substrate equilibrium binding—seem also to be modulated by membrane fluidity ^53^.

It is not completely understood how lipids can perturb P450 spin equilibrium ^48^, but the observed differences prove the intrinsic kinetic peculiarities of each system used to test chemicals. Molecular dynamic (MD) simulations and H/D exchange studies on CYP3A4 have shown that the interaction with the membrane occurs through specific lipid-protein interactions ^25, 55^ and is able to affect the opening/closing of the access tunnel ^48^, confirming experimental evidence obtained two decades before ^56^. Computational MD studies have also elucidated how phospholipids can induce an opening of membrane-facing tunnels in CYP1A2 ^57^. The speculated mechanism relies on the ability of the upper part of the TM helix to interact with a proline-rich segment of the catalytic domain that along with the FG loop is immersed in the membrane ^57^. This gave rise to several access channels from both solvent and membrane-facing tunnels. Similar studies conducted on CYP2C9 revealed the opening of additional access tunnels in the presence of membrane ^58, 59^. MD simulation performed by Fishelovitch *et al*. ^60^ has shown that the FMN binding domain of CPR regulates the water channel of CYP3A4 because of an overlapping of specific residues. When the FMN domain of CPR binds to CYP3A4, the water channel fully opened up, thereby allowing a flow of water molecules into the active site. However, the absence of membrane in this computational study raises the question of whether this conformational flexibility also occurs in the presence of membrane.

Though experimental demonstrations are essential, the computational studies have provided useful information, as well as quantitative thermodynamic parameters, that may potentially explain several observations in P450 kinetics. Nevertheless, given the importance of substrate availability and disposition for drug-metabolizing enzymes, there is a lack of compelling experimental evidence that can fulfill the requirement of detailed molecular descriptions. If structural x-ray crystallography has laid the groundwork for determining enzyme channels and quantifying active site volumes ^4^, it is still unable to look beyond the polypeptide architecture. On the other hand, recent progress in the use of solution- and solid-state NMR techniques is opening the way to a broader inspection of membrane-associated phenomena. Particularly, the high-resolution dynamics of membrane-bound P450 in the presence and absence of ligands to be measured from NMR will be highly valuable in fully understanding the function of P450.

## Current challenges and future developments

Membrane protein structure determination is in general an extremely challenging task because of the lack of stability of the protein outside its native environment. In cytochrome P450, the presence of interactions with the reducing counterparts, as well as the pivotal role of membrane in catalysis, adds fascinating complexities to the ongoing efforts. Given the limitations of the x-ray structural characterization discussed above, NMR—both solution- and solid-state approaches—is an excellent alternative for the structural and dynamic studies on cytochrome P450. As demonstrated for *b _5_* and its interactions with CYP2B4, NMR offers well-tested and sophisticated tools to probe MDs over a wide range of timescales. Through NMR, motions from nanosecond to millisecond timescales can be probed. Nevertheless, the overall size of the membrane-protein complex is still the main drawback in terms of spectral resolution; the reduced molecular mobility of proteins embedded in a membrane environment also contributes to poor sensitivity and resolution for traditional solution NMR experiments ^6^. For cytochrome P450, a further complication dwells in the paramagnetic nature of the iron in the heme prosthetic group, which quenches NMR signals in the proximity of the active site. In the coming years, NMR will be able to overcome several of its intrinsic limitations by the introduction of labeling strategies, paramagnetic relaxation enhancement effects, ultrafast magic-angle spinning, and sophisticated pulse sequences. Also, reconstitution of P450 and its redox partners now can be performed in more reliable and versatile model systems, such as nanodiscs that are used for solution NMR studies and macrodiscs for solid-state NMR studies.

Many scientific questions regarding the P450 metabolon require answers because of the innumerable biological and pharmacological implications. The ongoing structural studies would offer valid tools and provide intimate insights into biological interfaces. Nevertheless, the authors consider that the combined use of complementary biophysical techniques and biochemical approaches would create unique avenues to successfully overcome the challenges in fully understanding the enzymatic function of a variety of P450s. The acquired knowledge derived from these studies will set an experimental platform to investigate other electron-transfer protein complexes, such as the mitochondrial electron transport chain ^61^ and the nitric oxide synthase complex ^62^, which share several biochemical similarities and complexities with the P450 metabolon.
